# Box scaling as a proxy of finite size correlations

**DOI:** 10.1038/s41598-021-95595-2

**Published:** 2021-08-05

**Authors:** Daniel A. Martin, Tiago L. Ribeiro, Sergio A. Cannas, Tomas S. Grigera, Dietmar Plenz, Dante R. Chialvo

**Affiliations:** 1 Instituto de Ciencias Físicas (ICIFI-CONICET), Center for Complex Systems and Brain Sciences (CEMSC3), Escuela de Ciencia y Tecnología, Universidad Nacional de Gral. San Martín, Campus Miguelete, 25 de Mayo y Francia, 1650 San Martín, Buenos Aires Argentina; 2 Section on Critical Brain Dynamics, National Institute of Mental Health, National Institutes of Health, Bethesda, MD 20892 USA; 3 Instituto de Física Enrique Gaviola (IFEG-CONICET), Facultad de Matemática Astronomía Física y Computación, Universidad Nacional de Córdoba, 5000 Córdoba, Argentina; 4 Instituto de Física de Líquidos y Sistemas Biológicos (IFLySiB-CONICET), Universidad Nacional de La Plata, 1900 La Plata, Buenos Aires Argentina; 5 Departamento de Física, Facultad de Ciencias Exactas, Universidad Nacional de La Plata, 1900 La Plata, Buenos Aires Argentina; 6 Consejo Nacional de Investigaciones Científicas y Técnicas (CONICET), Godoy Cruz 2290, 1425 Buenos Aires, Argentina

**Keywords:** Statistical physics, thermodynamics and nonlinear dynamics, Computational biology and bioinformatics, Neuroscience, Mathematics and computing, Physics

## Abstract

The scaling of correlations as a function of size provides important hints to understand critical phenomena on a variety of systems. Its study in biological structures offers two challenges: usually they are not of infinite size, and, in the majority of cases, dimensions can not be varied at will. Here we discuss how finite-size scaling can be approximated in an experimental system of fixed and relatively small extent, by computing correlations inside of a reduced field of view of various widths (we will refer to this procedure as “box-scaling”). A relation among the size of the field of view, and measured correlation length, is derived at, and away from, the critical regime. Numerical simulations of a neuronal network, as well as the ferromagnetic 2D Ising model, are used to verify such approximations. Numerical results support the validity of the heuristic approach, which should be useful to characterize relevant aspects of critical phenomena in biological systems.

## Introduction

Complex biological phenomena at all levels, including macroevolution, neuroscience at different scales, and molecular biology are of increasing interest. In some systems, the origin of such complexity has been traced to critical phenomena via models and theory^1–13^. Nonetheless, the connection between complexity and criticality still needs to be established carefully in each case. Among others, a very distinctive indicator of the presence of critical phenomena is the observation of an increase in the correlation length as a function of the size of the system under study^14–17^. Such observation exposes one of the hallmarks of criticality: a complex dynamics which lacks a characteristic scale^18,19^. Less evident but equally relevant is the fact that at criticality the only scales are the ones “imposed” from outside, i.e., the finite size of the system and the limited time of system observation.

In most physical systems, criticality can be studied through the variation of system properties as some external parameter (say temperature) is changed. However this kind of tuning is usually off-limits in biological systems. Alternatively, one can in principle establish the lack of an intrinsic scale by demonstrating the size-scaling directly, i.e., by studying the correlation function in systems of increasing size. While this can be done with relative ease in numerical studies, it is much harder to achieve in experiments^20^. This is especially true in biological systems, which in most cases can neither be cut in small pieces, nor can they easily be enlarged. The brain is a prototypical biological system for which critical dynamics has been suggested to hold the key to its core functions^3,4,6,12,13^. The spatial extent of the correlations for ongoing and evoked brain activity was recently reported to depend in a unique way on the size of the observation window^4,12,21,22^, as well as in other biological systems, such as proteins and cell organelles^7–9^. However, there has been no systematic analysis on whether and how system-size correlation scaling can be approximated by varying the size of an observation window (without changing the system’s size), as Binder did with the order parameter scaling^23^. We will refer to this latter approach as “box-scaling”, since it resembles the fractal box-counting algorithm^24^. The purpose of this article is to fill that gap, and characterize the kind of results that can be obtained when a box-scaling approach is used, as a function of system parameters and state. We show that the computation of a correlation function under box-scaling allows to discriminate critical from non-critical regimes in systems exhibiting critical behavior. Therefore, box-scaling can be used as a proxy of finite size scaling on experimental setups where system size cannot be varied, such as in several biological systems.

The article is organized as follows. First the connected correlation function (CCF) is defined. Then the CCF is studied for a neuronal network model under two scenarios: in the first one we proceed in the standard manner, increasing the system size and determining its correlation behavior. In the second setting, the CCF is examined using a fixed system size (relatively large) while varying the size of the field of view (changing the observable window size). Motivated by currently available experiments on neuronal dynamics, we focus here on the case where the window is much smaller than the system size. After that, similar calculations are described in the ferromagnetic 2D Ising model. Finally, the collapse of all the CCF’s after rescaling is demonstrated for the two models. The article concludes with a discussion on the potential application of box-scaling to biological data, and a summary of the main results.

## Connected correlation function

The connected correlation function measures how a local quantity loses spatial correlation as distance is increased^25^. Two important quantities are usually extracted from this function, the decay with distance and the correlation length. Following previous work^4,7,8,10,12,17,21^ we compute it as,

1 $$\begin{aligned} C(r)= {1 \over c_0} { \sum _{i,j} \delta v_i \delta v_j \delta (r-r_{ij}) \over \sum _{i,j} \delta (r-r_{ij}) } \end{aligned}$$

where $\delta (r-r_{ij})$ is a smoothed Dirac $\delta$ function (we have taken $\delta (x)=H(x+0.5) H(0.5-x)$, where H is the Heaviside function), selecting pairs of signals (i.e., pairs of spin values, neuron states, or some other position dependent variable) at mutual distance *r*; $r_{ij}$ is the Euclidean distance from the site *i* to site *j*; $\delta v_i$ is the value of the signal $v_i$ of site *i* at time *t*, after subtracting the overall mean of signals $V(t)= (1/N)\sum _i^{N} v_i(t)$, i.e., $\delta v_i(t)=v_i(t)-V(t)$; ${1 \over c_0}$ is a normalization factor to ensure that $C(r=0)=1$. Notice that, potential spurious long-range correlations introduced by common external fields or perturbations, are not entering in *C*(*r*) of Eq. (1) since *V*(*t*) is the *instantaneous* space average. This also implies that the correlation length can be directly calculated as the zero crossing of the function.

Equation (1) is defined as the *space-averaged connected correlation function*, which differs from the *time-averaged connected correlation function* used to describe correlations between observables measured at different points in space,

2 $$\begin{aligned} C_{time}(r)= { \sum _{i,j} (v_i -\langle v_i \rangle ) (v_j -\langle v_j \rangle ) \delta (r-r_{ij}) \over \sum _{i,j} \delta (r-r_{ij}) } \end{aligned}$$

where $\langle v_i \rangle$ represents an average of $v_i$ over sufficiently long times (see e.g.^26^). In this case, the values of the correlation length and the spatial decay are determined by regression and fitting^27^. Thus, there is a subtle but important difference between Eqs. (1) and (2): the fluctuation $\delta v_i$ in *C*(*r*) is the difference between the local value of the signal $v_i$ and its instantaneous average over all the sample *V* at time *t*, while in $C_{time}$, fluctuations are computed with respect to time average $\langle v_i\rangle$. It is straightforward to demonstrate (at least for equilibrium systems, for long time series, without external perturbations) the equivalence between both quantities^10^,

3 $$\begin{aligned} c_0 C(r) = C_{time}(r) - \left\langle [ V - \langle V \rangle ]^{2} \right\rangle , \end{aligned}$$

i.e., they only differ by a constant. Thus, for experimental purposes averaged connected correlation function, Eq. (1) is preferred. To our knowledge, it was first used in^28^, in the context of bird flocks, and was later used in^4,7,8,12,17,22^, among others. Further details can be consulted in^10^.

The correlation length $\xi$ measures the scale at which two points start to become uncorrelated. In a critical system the correlation length is infinite, meaning that the decay of the correlation lacks a characteristic scale. In this case, the position of the zero of *C*(*r*) in Eq. (1) is a useful length scale, because then it increases proportionally to the system size *L* (the CCF defined with instantaneous space averages subtracted *must* have a zero^10^). This functional dependence, attesting scale invariance, suggests the presence of critical dynamics. However, it can not be used when the system size is fixed, as in the case of brain networks. Instead, we can subdivide the system in boxes of side *W*, (with $W < L$), and define a *characteristic scale* $r_0$ by the first zero of $C_W(r)$, where $C_W(r)$ is the CCF, Eq. (1), computed for partial regions of the entire system, of size *W*. Thus, the implementation follows the same logic and limitations than the box-counting algorithm commonly used to compute the fractal dimension of a data set, image or object^24^.

The hypothesis tested here is that the behavior of $r_0$ when *W* is varied with *L* fixed is the same as would be obtained with $W=L$ and varying *L*, at least when $W\ll L$ , namely

4 $$\begin{aligned} r_{0} \sim {\left\{ \begin{array}{ll} \xi \log (W/\xi ), &{} \xi \ll W < L,\\ W, &{} \xi \gg L\gg W. \end{array}\right. } \end{aligned}$$

This behavior can be justified for physical systems in equilibrium by extending the arguments of (Sect. 2.3.3 of Ref. 10) to the box-scaling case (see Supplemental Material). In the cases we show next, we find that the relations in (4) hold for all *W* up to $W \simeq L/2$.

In the following, we will study the scaling behavior of the characteristic length $r_{0}$ in two models: a 2D neuronal network, and the 2D ferromagnetic Ising model. In all simulations, *C*(*r*) and $C_{W}(r)$ were measured for all integer values of *r*. Subsequently, the smallest value of *r* for which *C*(*r*) [$C_{W}(r)$] is negative, $r_m$, was computed, and $r_{0}$ was found as the zero crossing of the linear fit of *C*(*r*) [$C_{W}(r)$] between $r=r_{m} -1$ and $r=r_{m}$.

## 2D Neuronal network model

We study a neuronal network described previously^29,30^. In short, the model is a cellular automata network on a square lattice, in which each neuron can be in one of three states at each time step: 0 for resting, 1 for active (lasting one time step), and 2 for refractory (lasting two time steps). Each neuron connects to *K* other neurons (here, $K=16$ always), in which Euclidean nearest neighbor neurons are favored by an exponential decaying function of the distance *r* between them (fixed here to $r_{d} = 5$). To ease the interpretation we imposed a cutoff in the interaction probability by preventing neurons to be connected at distances *r* greater than a given value (called here interaction length *I*, fixed here at $I=20$).

The network overall rate of activity is set by a very small ($h = 10^{-7} step ^{-1}$) independent Poisson perturbation to each neuron. We have verified that the results are robust over a wide range of *h* values (e.g., $h=10^{-9}$ to $10^{-4} step ^{-1}$). The model includes a control parameter $\sigma = K \times T$, in which *T* is the probability that an active neuron (i.e., in state 1) can excite one of the *K* neighbors that are connected to it. Therefore, as shown previously^29^, for any given value of *K*, the model can be made critical by changing the transmission probability *T* such that $\sigma \simeq 1$. We study two different scenarios: in the first we compute the CCF of the neuronal activities collected from systems of increasing sizes, from $L=40$ up to $L=640$. In the second case, a system of (fixed) large size ($L=1000$, i.e., $L \times L$ neurons) was simulated, from which the activity of each neuron inside of windows of sizes smaller than *L* (from $W=40$ up to $W=640$) were extracted for the correlation analysis. Since we are interested on situations where system size, although finite, is much larger than window size, we considered periodic boundary conditions. Results presented below correspond to averages of twenty or more realizations lasting $2.5 \times 10^{4}$ steps for each parameter value.

There are four scale lengths to consider in this model: The first is the interaction length *I*, it is the scale at which neurons interact via direct connections. The second is the system size *L*. The third is the size of the observation window ($W < L$) which determines the subset of neurons selected to compute the CCF. The last scale is the characteristic length $r_0$ which will be determined from the CCF.

**Figure 1:**
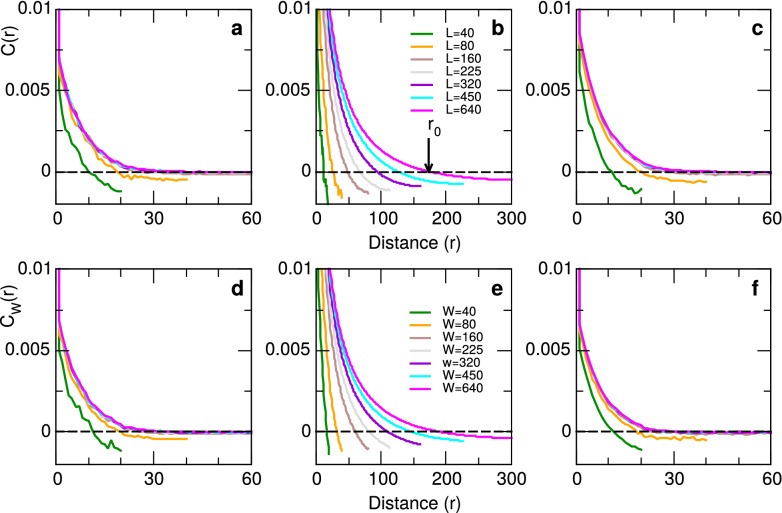
Connected correlation function of the neuronal network model. Curves in panels (**a**–**c**) for different system sizes *L* and those in (**d**–**f**) for different window sizes *W* computed on a system of size $L=1000$. Results are for three control parameter values corresponding to sub-critical ($\sigma$ = 0.64, panels **a**,**d**), critical ($\sigma$ = 1.024, panels **b**,**e**) and super-critical ($\sigma = 1.6$, panels **c**,**f**) regimes of the model. Arrow in panel (**b**) illustrates the value of $r_0$ for $L=640$.

Figure 1 shows the connected correlation functions computed for various system and window sizes, considering $v=1$ in Eq. (1), if neuron is active or refractory, and 0 otherwise, and three values of the control parameter $\sigma$. Computation of Eq. (1) was used for all pairs of neurons, taking one snapshot every 100 time steps, on the last 80% of the data (i.e., after 5000 equilibration steps). Only one window of each size was considered at each snapshot. Similar results can be found if Eq. (1) is calculated only for pairs of neurons on the same line, taking one snapshot at each time step (not shown). The results correspond to representative values of the control parameter: sub-critical $\sigma =0.64$, super-critical ($\sigma =1.6$) and critical ($\sigma \sim 1$) as indicated in the legend. Top panels (a, b and c) correspond to computations from increasing system sizes. Bottom panels (d, e and f) correspond to CCF computed using various window sizes from a system of size $L=1000$. It can be seen that the functions obtained when changing system size *L* or changing window size *W* are qualitatively very similar. In particular we note that, as expected from (4), for both sub-critical and super-critical values of $\sigma$ (panels a, d and c, f respectively) correlations do not grow much beyond the model interaction length $I=20$. At criticality, on the other hand $r_0$ increases when either the system itself (panel b) or windows increase in size (panel e).

**Figure 2:**
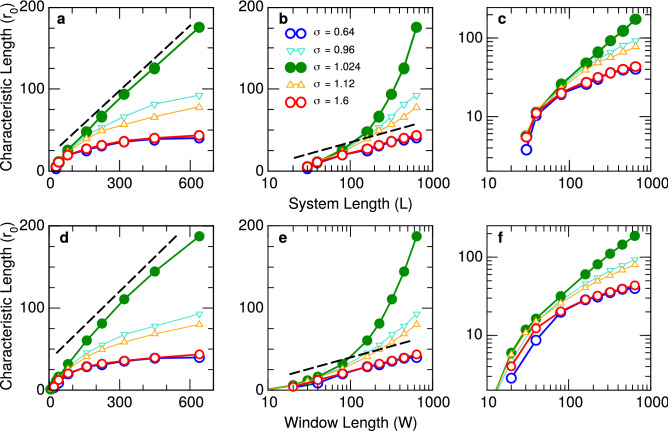
Characteristic Length $r_0$ of the neuronal network model. The zero crossings of the CCF shown in Fig. 1 are plotted in linear–linear (left), log–linear (middle) and log–log (right) axis. Top three panels correspond to different system size *L* and bottom three panels to different box length *W*. Different symbols correspond to the values of the control parameter $\sigma$ denoted in the legends. Dashed lines are visual aids to emphasize the predicted logarithmic behavior for both sub-critical and super-critical regimes (open circles) and the linear dependence expected for the critical regime (filled circles). Open triangles are used to denote results obtained for intermediate values of $\sigma$. In all cases, error bars are omitted, since error estimates are smaller than symbol size.

The values of $r_0$ extracted from the curves in Fig. 1 (as well as two other $\sigma$ values) are plotted in Fig. 2. Here the same data is presented using different axis formats to visualize the different functional dependency near and away the critical point. Dashed lines in panels b and e are a guide to the eye illustrating the expected logarithmic behavior of $r_0$ for sub-critical and super-critical regimes (open circles). The dashed lines in panels a and d denote the linear dependence expected for $r_0$ (filled circles) in the critical regime. Finally, the same data is plotted in log-log axis in panels c and f to reveal the crossover behavior for *W* values close to and smaller than the interaction length ($I=20$), denoted by the deviation from the asymptotic linear dependency for large *W*. Also it is worth to notice the small deviation of the linear scaling observed at criticality when the value of *W* approaches the system size *L* ($W=640$ in panel d of Fig. 2). This deviation is expected from the theory, as discussed in the Supp. Material. In brief, $r_0$ depends on both *W* and *L*. If $W \ll L$, then $r/L \ll 1$ and the dependance of $r_0$ on *L* does not need to be considered. On the other hand, for small *L*, $r_0(W,L)$ grows with *L*, for fixed *W*, having it’s minimum for $W=L$.

Overall, these results show that the scaling of the characteristic length $r_0$ follows a similar functional dependence with either the box-scaling or the system-size.

## 2D ferromagnetic Ising model

The results obtained from the neuronal model were replicated in numerical simulations of the ferromagnetic 2D Ising model on a square lattice, using periodic boundary conditions, with Hamiltonian $H=-\sum _{\langle i,j \rangle } s_i s_j$, where $s_i = \pm 1$ and ${\langle i,j \rangle }$ stands for sum over nearest-neighbors. Similar to the previous model, the simulations used two scenarios: in the first the CCF was computed in the standard way from a model running on square lattices of increasing sizes from $L=16$ up to $L=512$. In the second setup, a relatively large $L=600$ square lattice (i.e., $600 \times 600$ spins) was simulated, and the CCF was computed from square window of smaller sizes from $W=16$ up to $W=512$. Results are computed using $v=s$ in Eq. (1). They correspond to averages of five realizations each one lasting at least $5\times 10^{6}$ Monte Carlo steps, under the same conditions as in the neuronal model (the first $20\%$ time steps are not considered, after that, one snapshot is taken every 100 MC steps, all pairs of spins are considered in Eq. (1), and only one window of each size is taken). In all cases we considered periodic boundary conditions. Similar results can be found using open-boundary conditions, see the Supp. Material.

**Figure 3:**
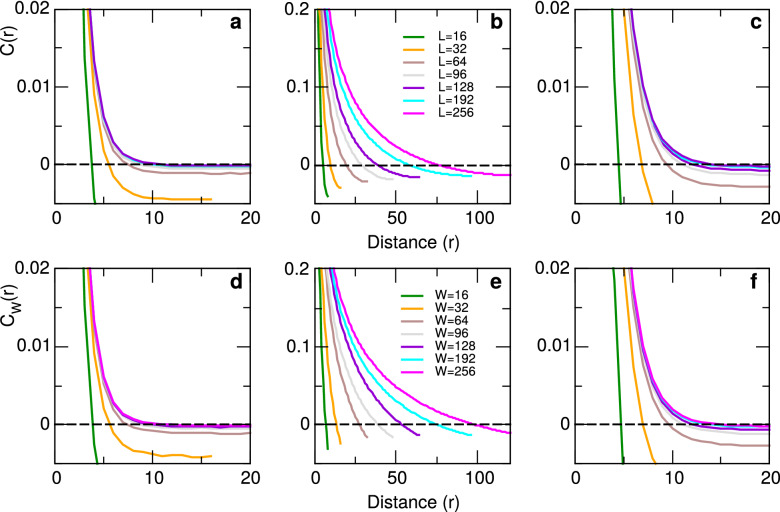
Connected correlation function for the ferromagnetic 2D Ising model. Typical results for three temperatures $T =2.00$ (panels **a**,**d**); $T=2.27$ (panels **b**,**e**) and $T= 3.0$ (panels **c**,**f**) and various lattice and window sizes. Curves in the top panels computed from different system sizes *L* and those in the bottom panels computed on a system of size $L=600$ using the window sizes *W* indicated in the legend.

Figure 3 shows representative results for the two scenarios at three different temperatures: sub-critical ($T=2.0$, Panels a and d), critical ($T=2.27$, Panels b and e) and super-critical ($T=3.0$, Panels c and f). The top panels represent results computed for increasing system sizes and the bottom panels for a fixed lattice size and various window sizes. Note that, as already seen in the simulations of the neuronal model, the computation of the CCF by changing system size *L* or by changing window size *W* produces very similar results.

**Figure 4:**
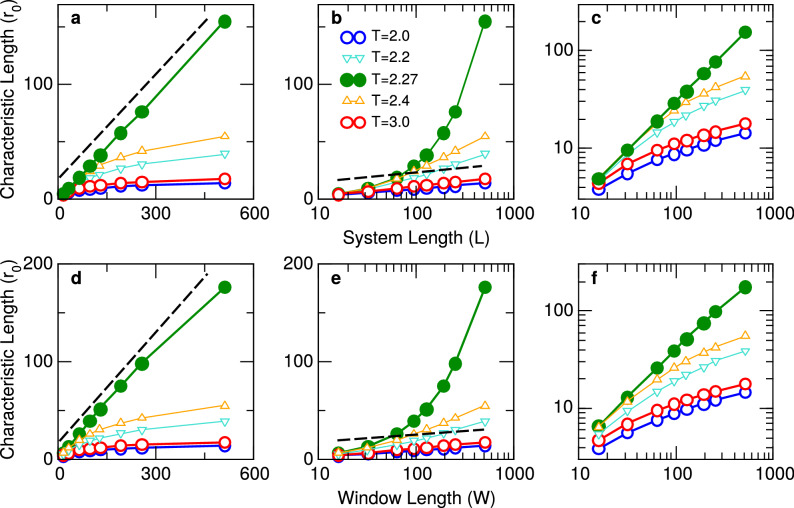
Characteristic length $r_0$ of the ferromagnetic 2D Ising model. The zero crossings of the computed CCF are plotted in linear–linear (left), log–linear (middle) and log–log (right) axis. Top three panels correspond to different system sizes *L* and bottom three panels to different window lengths *W*. Different symbols correspond to the values of the temperature *T* denoted in the legends. Dashed lines are visual aids to emphasize the predicted logarithmic behavior for both sub-critical and super-critical regimes (open circles), and the linear dependence expected for the critical regime (filled circles). Open triangles are used to denote results obtained for intermediate values of temperature indicated in the legend. In all cases, error bars are omitted, since error estimates are smaller than symbol size.

The dependence of $r_0$ with system and window size is shown in Fig. 4 using the same format as in Fig. 2 for the neuronal model. It is clear that the results obtained from varying the system or the window size show a striking similarity, suggesting that for this system the approximation is also valid. Notice the small deviation from linearity observed at criticality for $W=512$ in panel d of Fig. 4 which is similar to that exhibited by the neuronal model for *W* sizes near the value of *L*.

## CCF rescaling and data collapse

The box-scaling analysis presented here can be considered as a variant of the finite-size scaling (FSS) method^15,19,20,31,32^ used extensively in a broad range of situations, including dynamic parameters such as viscosity or coalescence point (see for instance^33,34^), or time decaying functions (i.e., dynamic scaling^35,36^).

**Figure 5:**
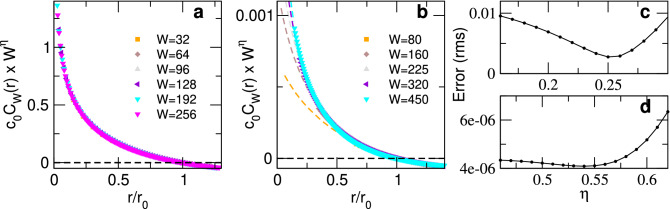
Correlation collapse after rescaling for the ferromagnetic 2D Ising and the neuronal model at criticality. Panel (**a**) 2D Ising model rescaled CCF as a function of *W* with fixed *L* (same same data as in Fig. 3e, with $L=600$, $W< L$, $T=2.27$), collapsed as $c_0 \, C_{W}(r) \times W^{\eta }$ v.s. $r/r_0$, with $\eta =0.25$. Panel (**b**) Collapse for neuronal model, using exponent $\eta =0.54$. For each value of *W*, points with $r<I$ are shown with a dashed line of the same color. Panels (**c**,**d**) relative collapse error as a function of critical exponent $\eta$ for the Ising and the neuronal model, respectively. All simulation parameters are as in previous figures.

FSS often allows to expose the scale invariance observed close to a critical point by the successful collapse of some observable, using the system size, the correlation length, and some universal critical exponents^20^. In analogy with FSS, using box-scaling we show in Fig. 5, the collapse of $c_0 C_W(r) W^{\eta }$ as a function of $r/r_0$ for both Ising and neuronal model (see the Supp. Material for a derivation). For the Ising model, we find that the best collapse is for $\eta \simeq 0.25$ in accordance with results for standard FSS collapse.

For the neuronal model, we lack of any previous estimate of $\eta$. Empirically, we found that $\eta \simeq 0.4-0.7$ gives good collapse results for $C_W(r)$ in the range of $r>I$. For distance values $r<I$ collapse of $C_W(r)$ is not expected, since within this range the correlations are dominated by the interactions rather than by the collective phenomena. Finally, results for $W=640$ were not considered since the condition $W\ll L$ is not fulfilled. The critical exponent values used to rescale were chosen based on the minimization of the relative collapse error (Fig. 5c,d), defined in the Supp. Material. Notice that for both cases, the error is two orders of magnitude smaller than the observable values.

While it is not the main focus of this work, scaling and collapse can be demonstrated for other observables, including magnetization $|\langle m \rangle |$, where $m(t)={1\over W^{2}} \sum _i s_i(t)$, and its related susceptibility, $\chi = {W^{2}\over T} [\langle m^{2}\rangle -\langle m\rangle ^{2} ]$ in the Ising model. Box-scaling data can be collapsed using the same values of critical exponents $\nu =1$, $\beta =1/8$ and $\gamma =7/4$ used in FSS, just replacing system size *L* by window size *W* (see Supp. Material). Analogous results can be obtained for the neuronal model (see Supp. Material). It need to be noted that the formal connection between the box-scaling studied here and FSS could be traced back to Binder’ work^23^ who described spin distributions, fluctuations and other observables in the equilibrium Ising system as a function of window size.

## Discussion

We presented results for a *correlation function* well suited for out of equilibrium experiments. We have studied the behaviour of the CCF as a window length is varied for two models: the Ising model, which represents an equilibrium material, and it’s dynamics are governed by a Hamiltonian, and the neuronal network model, which represents (a piece of) living matter, whose elements are cellular automatons, and their dynamics may be modified by external perturbations. Both models present critical dynamics and in both cases, box-scaling reproduces finite-size scaling results. The most relevant difference between them, in the context of box-scaling, is that in the Ising model, interaction length is much smaller than any considered window, while it is smaller but comparable to smallest windows for the neuronal model. This difference is noticed, for instance, from the bending of $r_0$ v.s. *W* in Fig. 2.

Regarding the feasibility of box-scaling on experimental setups, where system size cannot easily be varied, it needs to be noticed that the method is affected by the same limitations as other approaches for biological data: inhomogeneities, non-stationarity, and, in most cases, finite length of the data. However, we should stress that this approach has already been shown to be practical in some settings^4,7,8,12,22^. In Supp. Material (see Supp. Fig. 4) we reproduce results for characteristic length as a function of window size from already published^12^ human fMRI data (where the already mentioned limitations are present), and we find that critical dynamics is unambiguiously distinguished from sub or super critical, using the same scaling as in Fig. 2d–f of Fig. 4d–f.

Relatively small windows may be affected by inhomogeneities such as the local fluctuations in the number of elements (i.e., neurons). This effect is present on a variety of situations whenever a portion of a system is assumed to represent the whole. While it may not be easy to account for all inhomogeneity effects, it is straightforward to estimate it’s contribution. For instance, several small non-overlapping windows may be taken from the largest available window, and box-scaling analysis can be performed on each of them, measuring to what extent the result on one of them is representative of the whole group.

Regarding non-stationarity, it should be stressed that box-scaling computes the space-averaged connected correlation function, where the instantaneous average is subtracted to each signal. This correlation function differs from the more frequently used time averaged correlation function, or the Pearson correlation (where time averages are subtracted). Thus the calculation can be performed independently on different snapshots, and box-scaling analysis can be performed as a function of time.

Indeed, box-scaling does not require a *large* amount of data: as an example, we have successfully reproduced the results for the Ising model using $1\%$ of the data shown in Figs. 3 and 4 (see Supp. Material), entailing few hundreds of samples. The reason behind this is that system state is not derived by a single value of $r_0$ (which may be noisy in some experimental conditions), but it is derived from the whole $r_0(W)$ curve, which shows a linear/logarithmic dependence for systems at/away from criticality. Moreover, the scale-invariance typical of criticality was well captured by the data collapse after rescaling demonstrated in Fig. 5, remarking that at odds with zero-crossing, the collapse takes information from all recorded data points.

Finally, we would like to point out that an analysis similar to box-scaling method has recently been proposed in the context of scale-free networks^37^. There, a scaling hypothesis for finite size scale-free networks is proposed and tested over several naturally occurring networks.

In summary, the results obtained from a neuronal network model and from the ferromagnetic 2D Ising model show that the finite-size scaling of the correlation length $\xi$ can be approximated—near the small-size limit—by the dependence of the characteristic length $r_0$ on window size. Results are derived from CCF where instantaneous averages are considered, in such way that the result is insensitive to inhomogeneous distribution of signals or external perturbations on the whole window. The results are particularly relevant at the experimental level in neuroscience, in which techniques to map different areas of the brain cortex are now available^38^, while changing system size is not feasible. In that direction, the present analysis is fully consistent with the experimental observations being reported using optogenetic tools^21^ and also multi-electrode arrays^22^. It should also be straightforward to apply box-scaling to in vitro results, where both system and window sizes may be changed^39^.

## Supplementary Information

Supplementary Information.
