# Supplementary material for: Box scaling as a proxy of finite size correlations
Source: Sci Rep. 2021 Aug 5;11:15937. doi: 10.1038/s41598-021-95595-2 (PMC8342522; doi:10.1038/s41598-021-95595-2)
Supplement: Supplementary file 1 — Supplementary Information. [file 41598_2021_95595_MOESM1_ESM.pdf]

Supplemental Material of the manuscript: Box scaling as a proxy of finite size correlations

Daniel A. Martin, Tiago L. Ribeiro, Sergio A. Cannas, Tomas S. Grigera, Dietmar Plenz and Dante R. Chialvo

BOX SCALING IN EQUILIBRIUM

Characteristic Length behavior: The relation between r_0 and W observed in our numerical experiments can be understood in the case of a physical system in equilibrium, where one can relate the connected correlation function (CCF) computed with space averages (as we do here) to the usual *time-averaged connected correlation function*. We show here how to adapt the argument of [1, Sec. 2.3.3] to the case of $W < L$ in a system of d dimensions. We start with the relation between $C_W(r)$ and the correlation $C_{time}(r)$, which is[1]:

$$c_0 C_W(r) = C_{time}(r) - \left\langle [V - \langle V \rangle]^2 \right\rangle. \tag{1}$$

The variance of V computed over a volume W^d can be written in terms of $C_{time}(r)$,

$$\left\langle [V - \langle V \rangle]^2 \right\rangle = \frac{1}{W^d} \int_{W^d} d^d r\, C_{time}(r) g(r), \tag{2}$$

where $g(r)$ is the radial distribution function describing the density correlations of the lattice, and arises here because the definition of the CCF includes a denominator which is essentially $r^{d-1} g(r)$. The definition of r_0 is $C_W(r_0) = 0$, so that

$$C_{time}(r_0) = \frac{1}{W^d} \int_{W^d} d^d r\ C_{time}(r) g(r). \tag{3}$$

This equation is useful because, for equilibrium physical systems near a critical point, we know the scaling form of $C_{time}(r)$, which we can use to obtain the relationship we seek.

We must distinguish two cases:

(i) $\xi \ll W < L$: In this case we can write $C_{time}(r) = r^{-d+2-\eta} e^{-r/\xi}$. For large r, the system is homogeneous, and we can approximate $g(r) \approx 1$. Clearly r_0 will depend on ξ and W but not on L. Due to the short range of $C_{time}(r)$ the integral in Eq. 3 can be extended to infinity, so that

$$r_0^{-d+2-\eta} e^{-r_0/\xi} = \frac{1}{W^d} \xi^{2-\eta} \int^{\infty} dx\, x^{1-\eta} e^{-x}, \tag{4}$$

which gives to leading order

$$r_0 \sim \xi \log(W/\xi). \tag{5}$$

(ii) $\xi \gg L \gg W$. This is the critical case, where $C_{time}(r) = r^{-d+2-\eta} h(r/L)$[2]. For $L \to \infty$, the scaling function $h(x)$ goes to a constant and the decay is a pure power law, but for finite L the decay is modulated by the scaling function. Plugging into Eq. 3 and using again $g(r) \approx 1$ we get

$$r_0^{-d+2-\eta} h(r_0/L) = W^{-d} L^{2-\eta} \int^{W/L} h(u) u^{1-\eta}\, du. \tag{6}$$

If $W = L$ the integral reduces to some constant, and we see that $r_0 \sim L$ is a solution, justifying the claim that the zero of $C_W(r)$ is proportional to L when the correlation is computed over the whole sample. If $W < L$ (i.e. if $C_W(r)$ is computed over a box smaller than the whole system), then in general r_0 will depend on both L and W. However if $W \ll L$ we are in a regime where $C_W(r)$ should decay almost as a pure power law, because the modulating effects of $h(x)$ will be noticeable only for $r \approx L$. This means that we can replace $h(u)$ with a constant inside the integral, so that

$$r_0^{-d+2-\eta} h(r_0/L) \sim W^{-d} L^{2-\eta} \int^{W/L} u^{1-\eta}\, du \sim W^{-d+2-\eta}, \tag{7}$$

which gives $r_0 \sim W$.

Re-scaling of the connected correlation function: For the 2D Ising model at criticality, correlations can be described as $C_{time}(r) = f(r/L) \times r^{-\eta}$, which can be rewritten as $C_{time}(r) = g(r/L, r/W) \times W^{-\eta}$. Also, $\langle [V - \langle V \rangle]^2 \rangle = \langle [m - \langle m \rangle]^2 \rangle = T\chi/W^d$, where χ is the susceptibility calculated on a window. When simulations are performed as a function of system size, $\chi_L \propto L^{\gamma-d}$, where γ is a critical exponent, and χ_L is the standard susceptibility, measured over all the spins of a system of size L. For the 2D Ising model, $\gamma = 7/4$. When calculations are performed as a function of window size W, we find that χ follows the same behavior as χ_L, both at and away from criticality, see Supp. Fig. 1. In particular, at criticality, $\chi \propto W^{\gamma'-d}$ with $\gamma' \simeq \gamma$ (this can also be observed from the rescaling of m and ξ at criticality, Supp. Fig. 5). We remark that, despite this similarity rigorous results proving the $\gamma' = \gamma$ relation, for the Ising or any other model, are lacking. Considering Fisher scaling relation[3] $\eta = 2 - \gamma/\nu$, and $\nu = 1$ for Ising model with $d = 2$, we get

$$c_0 C_W(r) = h(r/L, r/W) W^{-\eta},$$

where h is some smooth function. So, for $r \ll L$, $c_0 C_W(r) \times W^{\eta}$ should collapse into a single curve as a function of r/r_0 (see Fig. 5 in main text). Since our estimate of ν for the neuronal model (see Supp. Fig. 5) is close to 1, the same rescaling was attempted for that model (shown in Fig. 5 of main text).

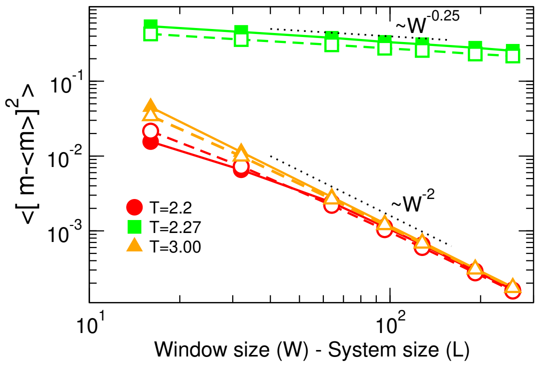

Figure 1. Scaling of magnetization fluctuations for the ferromagnetic 2D Ising model: Magnetization fluctuations $\langle [m - \langle m \rangle]^2 \rangle$, calculated on windows of size W or systems of size L, as a function of W, or L, for subcritical (circles, $T = 2.2$), critical (squares, $T = 2.27$), and supercritical (triangles, $T = 3$) temperatures. Full lines and filled symbols stand for $W = L$, while dashed lines and empty symbols stand for $L = 600$ and variable W, lines with $\langle [m - \langle m \rangle]^2 \rangle \propto W^{\gamma - 2} = W^{-0.25}$ and $\langle [m - \langle m \rangle]^2 \rangle \propto W^{-2}$ have been added as a guide to the eye. Other simulation parameters as in Fig. 3 of the main text.

METHOD' ROBUSTNESS

To account for potential concerns about the robustness of our calculations, we reproduced the procedure described in the main text for the 2D Ising model, for two alternative cases. First we recalculated $C_W(r)$ on relatively short simulations, using only 1% of the data presented in the main text. Second, we replicated the calculations for a system with open–boundary conditions. As described in the next paragraphs, in both cases, we find results that are very similar to the results reported in the main text, obtained with periodic-boundary conditions and relatively long simulations. Finally, we have plotted the correlation length as a function of window size, extracted from already published fMRI data[4] on human brains, where typical limitations of experimental setups are present.

Results with relatively short statistics: We have repeated simulations and computation of the connected correlation function for the ferromagnetic 2D Ising model as a function of W (Fig. 3 d-f of main text), using less data than in the results presented in the main text. Results are shown in Supp. Fig. 2a-c. Using this data, we computed the zero crossings of the CCF, and plotted them in different axis scales, in Supp. Fig. 2d-f. Simulations were performed for 5 samples per temperature, considering one window of each size for each sample. Simulations lasted 50.000 MC steps, and results where taken once every 100 MC steps (i.e. 500 system snapshots where used for calculation). This is 1% of the simulation time shown in main text. We can see from Supp. Fig. 2 that, although results are noisier, the behavior at the critical point is still clearly distinguishable from the deeply sub/supercritical regime, and also from the slightly sub/supercritical one. Similar results where found for Neuronal model (not shown).

Results with open–boundary conditions: We have repeated the simulations of Fig. 3 d-f (connected correlation function for the ferromagnetic 2D Ising model as a function of W) of main text, using open–boundary conditions, and all other parameters as in the main text. The window, of size $W < L$ is centered with the system. The results shown in Supp. Fig. 3 are very

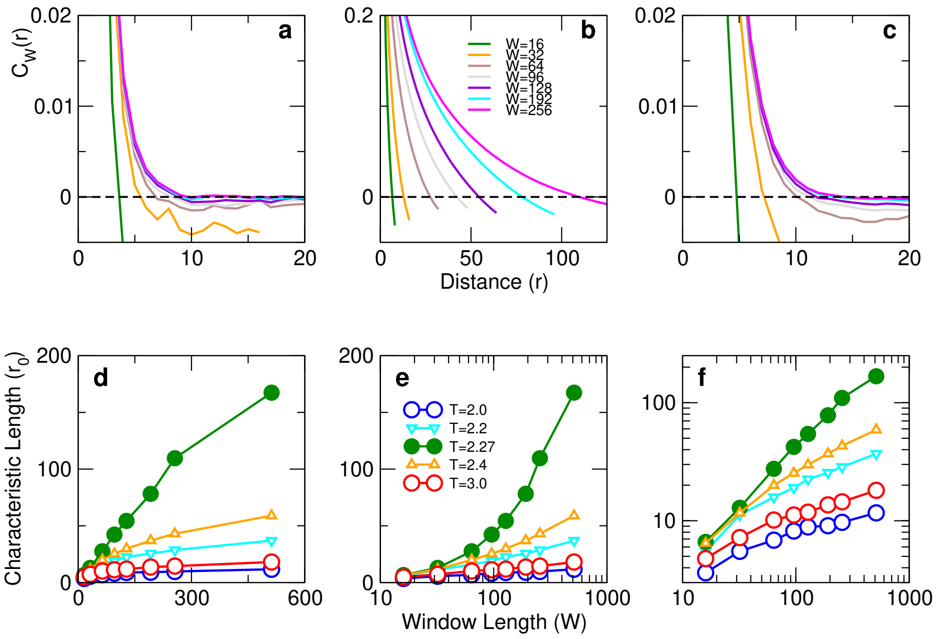

Figure 2. Connected correlation function, and characteristic length r_0 for the ferromagnetic 2D Ising model computed from 1% of the data points used in the results presented in the main text. Panels a-c: Typical results for three temperatures $T = 2.00$ (panel a); $T = 2.27$ (panel b) and $T = 3.0$ (panel c) and various window sizes, computed from 1% of the data used in Fig. 3 d-f of main text. Panels d-f: Characteristic length (r_0), computed using 1% of the data in Fig. 4 d-f of main text, in linear-linear (panel d), log-linear (panel e), and log-log (panel f) axis, for different values of temperature T, denoted in the legends. All simulations parameters are as in Fig. 3 and 4 of the main text.

similar to those obtained using periodic–boundary conditions. Identical behavior was obtained for the neuronal model using open–boundary conditions (data not shown).

fMRI results: We have reproduced already published human fMRI data[4], where the instantaneous connected correlation function is calculated over 35 clusters, of different sizes, and 8 resting state networks, in Supp. Fig. 4. Although the window sizes (computed here as the cubic root of the number of voxels) cover roughly one order of magnitude, and limitations common to experimental setups (finite time series, inhomogeneities and several sources of error) are present, it is straightforward to find, from linear-linear and linear-log plots, that results are compatible with critical regime.

ORDER PARAMETER AND SUSCEPTIBILITY BOX-SCALING

Order parameter and fluctuations: For the sake of consistency, here we illustrate how the finite-size behavior of other system' quantities are captured by the box-scaling approach. The results in Supp. Fig. 5 show the order parameter (i.e., magnetization $|\langle m\rangle|$, where $m = \frac{1}{W^2}\sum_i s_i(t)$) and the magnetic susceptibility, estimated as $\chi = \frac{W^2}{T}[\langle m^2\rangle - \langle m\rangle^2]$, as a function

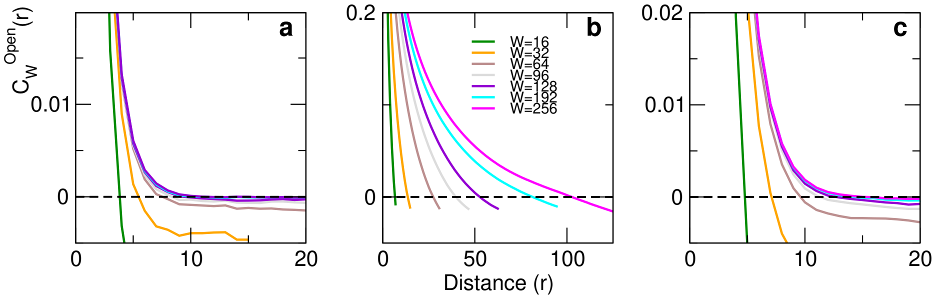

Figure 3. Connected correlation function for the ferromagnetic 2D Ising model with open–boundary conditions. Typical results for three temperatures $T = 2.00$ (panel a); $T = 2.27$ (panel b) and $T = 3.0$ (panel c) and window sizes, computed with open–boundary conditions. All simulations parameters are equal to the used in Fig. 3 of main text.

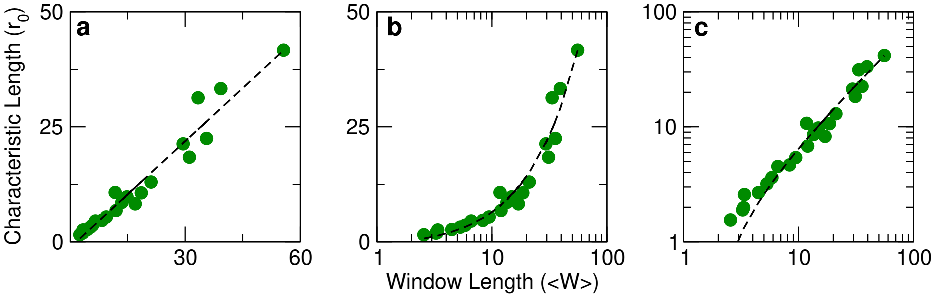

Figure 4. **Scaling of the characteristic length r_0 with window length for previoulsy reported fMRI experimental data**. Characteristic length as a function of window size is plotted in linear-linear (a), linear-log (b) and log-log (c) axis, where the symbols correspond to the zero crossings of the CCF and the dashed line to a linear fit ($r_0 = a + W \times b$ with $a = -2.5$, b=1.5 ± 0.07, correlation coefficient $r = 0.97$). Notice that a semilogarithmic scaling, which must be seen as a straight line in panel b, can be easily rejected. For completeness we show also the log-log plot in panel c. Window sizes and correlation lengths are measured in terms of voxel length, where each voxel represents a 2mm side cube of a standardized brain. Further details can be found in the original artice[4].

of temperature T, for T close to T_c, for system of $L = 600$, and several values of W (panels a and d respectively). It can be seen that the curves collapse on a single function after rescaling, in the same manner as when performed as a function of system size. Explicitly, we plot $|\langle m \rangle| \times W^{\beta/\nu}$, and $\chi \times W^{-\gamma/\nu}$ as a function of $(T - T_c) \times W^{1/\nu}$, in Fig. 5 panels b and e respectively, using the same values of critical exponents ($\nu = 1$, $\beta = 1/8$ and $\gamma = 7/4$) and critical temperature ($T_c = 2.2691$) as in the usual, system dependent collapse. In Supp. Fig. 5 panels c and f, we show relative collapse errors as a function of critical exponents, showing that above mentioned exponents are indeed reasonable values.

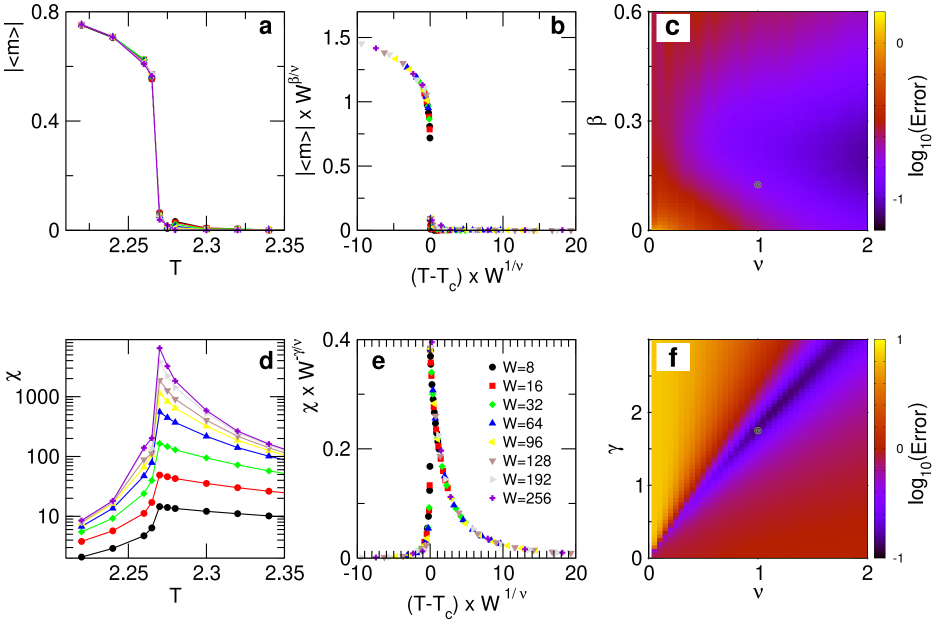

Figure 5. **Rescaling of order parameter and fluctuations for ferromagnetic 2D Ising model:** Panel a: Magnetization as a function of T for several values of W, with fixed L. Panel b: Collapse after rescaling of the data on panel a, using 2D Ising critical exponents ($\nu = 1$, $\beta = 1/8$). Panel c: Logarithm of the magnetization data collapse error as a function of ν and β. The green ring denotes the parameter values used in panel b. Panel d: Magnetic susceptibility χ as a function of T for several values of W, with fixed L. Panel e: Collapse after rescaling of the data in panel d, using Ising critical exponents ($\nu = 1$, $\gamma = 7/4$). Panel f: Logarithm of the susceptibility collapse error as a function of ν and γ. The green ring denotes the parameter values used in panel e. In all cases, $L = 600$ and all simulation parameters are as in the main text.

A data rescaling showing a collapse, is more challenging for the neuronal model and will require future work. The simple definition of order parameter as the density of active neurons seems insufficient to describe ordering, at least in the same sense that magnetization does it for the Ising model. In addition, neither the exact value of σ_c, nor the critical exponents (β, γ, and ν) are known beforehand. Moreover, most of the windows we consider are either a few interaction lengths long (i.e $W \sim 5I$) or large compared to system size ($W \sim L/2$). Despite these caveats, Supp. Fig. 6 shows the collapse after rescaling of the average fraction of active neurons, f, and its related susceptibility, $\chi_f = W^2\langle[f - \langle f\rangle]^2\rangle$ (for neuron model, $\langle\rangle$ represents average over long times). Based on error minimization, we have chosen the following rescaling parameters: $\sigma_c = 1.021$, and $\nu = 0.92$, $\beta = 0.87$, $\gamma = 0.17$. This values follow the scaling relation[3] $\nu d = 2\beta + \gamma$. However, they cannot be linked to correlation function collapse: the scaling relation $\eta = 2 - \gamma/\nu$, does not seem to hold (we had found $\eta \simeq 0.54$ in main text). Please note these results may be shifted by interaction length effects for small windows (there is a large I/W ratio), while for large W, results may be perturbed by a small system size (large W/L ratio).

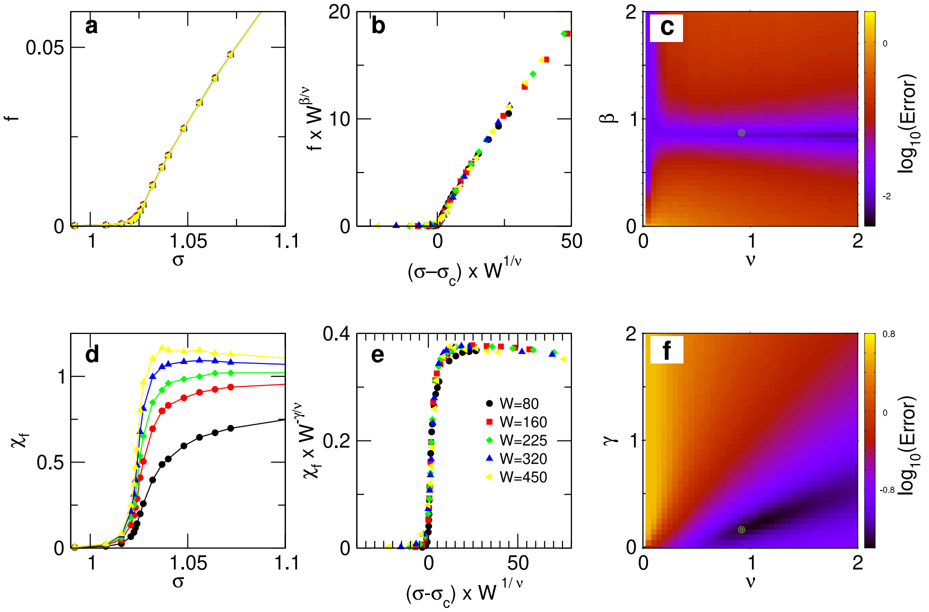

Figure 6. Box-scaling for order parameter and fluctuations for neuronal model: Panel a: Fraction of active neurons, f, as a function of σ for several values of W, with fixed L. Panel b: Collapse of panel a, after rescaling using critical exponents ($\sigma_c = 1.021$, $\nu = 0.92$, $\beta = 0.87$). Panel c: Logarithm of f collapse error as a function of ν and β. A green ring denotes values used in panel b. Panel d: Related susceptibility χ_f as a function of σ for several values of W. Panel e: Rescaling collapse of the results in panel d, using Ising critical exponents ($\sigma_c = 1.021$, $\nu = 0.92$, $\gamma = 0.17$). Panel f: Logarithm of susceptibility collapse error as a function of ν and γ. A green ring denotes values used in panel e. In all cases cases, $L = 1000$ and all simulation parameters are as in main text.

Rescaling' collapse error. In Fig. 5 on main text, collapse error was computed as the root mean square error. For each value of ν, each curve is rescaled. The set of points $\{r, C_W(r)\}$ is replaced by $y_w(x_i)$ (where $x = r/r_0$ and $y = c_0 C_W \times W^{\eta}$ in Fig. 5). For each x value, we calculate the average function: $y_{AVG}(x) = \frac{1}{N_c}\sum_{w=1}^{N_c} y_w(x)$, where $y_w(x)$ is calculated from the linear interpolation of the two nearest values of $y_w(x_i)$, and N_c is the number of curves. Error is then computed as the root mean square distance to this curve:

$$rms = \sqrt{\frac{\sum_{w,i}\left[y_w(x_i) - y_{AVG}(x_i)\right]^2}{N_p}}, \tag{8}$$

where N_p is the number of data points. A similar rescaling is performed in Supp. Figs. 5 and 6 (now $x = (T - T_c) \times W^{1/\nu}$). Notice that now, changing β or γ values produces $y \to \alpha y$ (for some α value), which artificially multiples rms error by α (and would lead to consider best collapse values as $\gamma = \infty$, $\beta = 0$). In order to correct this, in Supp. Figs. 5 and 6 we compute the

normalized rms error, $nrms = rms/||y_{AVG}||$, where $||y_{AVG}|| = \sqrt{\frac{\sum_{w,i} y_{AVG}(x_i)^2}{N_p}}$.

References

1. Cavagna A., Giardina I., Grigera T. S. The physics of flocking: Correlation as a compass from experiments to theory. *Physics Reports* **728**,1– 62, (2018).

2. Cardy, J. L. (Ed.) *Finite-size Scaling*. North Holland, Amsterdam, (1988).

3. Fisher M. E. Renormalization group theory: Its basis and formulation in statistical physics. *Rev. Mod. Phys.* **70**, 653–681, (1998).

4. Fraiman, D. & Chialvo, D. What kind of noise is brain noise: anomalous scaling behavior of the resting brain activity fluctuations. *Frontiers in Physiology* **3**, 307 (2012).
